# Dietary Sialyllactose Influences Sialic Acid Concentrations in the Prefrontal Cortex and Magnetic Resonance Imaging Measures in Corpus Callosum of Young Pigs

**DOI:** 10.3390/nu9121297

**Published:** 2017-11-28

**Authors:** Austin T. Mudd, Stephen A. Fleming, Beau Labhart, Maciej Chichlowski, Brian M. Berg, Sharon M. Donovan, Ryan N. Dilger

**Affiliations:** 1 Piglet Nutrition & Cognition Lab, University of Illinois Urbana-Champaign, Urbana, IL 61801, USA; amudd2@illinois.edu (A.T.M.); sflemin2@illinois.edu (S.A.F.); 2 Neuroscience Program, University of Illinois Urbana-Champaign, Urbana, IL 61801, USA; 3 Mead Johnson Pediatric Nutrition Institute, Mead Johnson Nutrition, 2400 W Lloyd Expressway, Evansville, IN 47712, USA; beau.labhart@mjn.com (B.L.); maciej.chichlowski@mjn.com (M.C.); dr.brianberg@gmail.com (B.M.B.); 4 Division of Nutrition Sciences, University of Illinois Urbana-Champaign, Urbana, IL 61801, USA; sdonovan@illinois.edu; 5 Department of Food Science and Human Nutrition, University of Illinois Urbana-Champaign, Urbana, IL 61801, USA

**Keywords:** neurodevelopment, milk oligosaccharide, sialyllactose, pig, corpus callosum, sialic acid, pediatric nutrition

## Abstract

Sialic acid (SA) is a key component of gangliosides and neural cell adhesion molecules important during neurodevelopment. Human milk contains SA in the form of sialyllactose (SL) an abundant oligosaccharide. To better understand the potential role of dietary SL on neurodevelopment, the effects of varying doses of dietary SL on brain SA content and neuroimaging markers of development were assessed in a newborn piglet model. Thirty-eight male pigs were provided one of four experimental diets from 2 to 32 days of age. Diets were formulated to contain: 0 mg SL/L (CON), 130 mg SL/L (LOW), 380 mg SL/L (MOD) or 760 mg SL/L (HIGH). At 32 or 33 days of age, all pigs were subjected to magnetic resonance imaging (MRI) to assess brain development. After MRI, pig serum and brains were collected and total, free and bound SA was analyzed. Results from this study indicate dietary SL influenced (*p* = 0.05) bound SA in the prefrontal cortex and the ratio of free SA to bound SA in the hippocampus (*p* = 0.04). Diffusion tensor imaging indicated treatment effects in mean (*p <* 0.01), axial (*p <* 0.01) and radial (*p* = 0.01) diffusivity in the corpus callosum. Tract-based spatial statistics (TBSS) indicated differences (*p <* 0.05) in white matter tracts and voxel-based morphometry (VBM) indicated differences (*p <* 0.05) in grey matter between LOW and MOD pigs. CONT and HIGH pigs were not included in the TBSS and VBM assessments. These findings suggest the corpus callosum, prefrontal cortex and hippocampus may be differentially sensitive to dietary SL supplementation.

## 1. Introduction

Appropriate nutrition early in life is essential to support optimal growth and developmental trajectories in the infant. Human milk contains a unique composition of bioactive components and is generally considered the gold standard for infant nutrition [1]. While breastfeeding is ideal, it is not always a viable option, thus infant formula is often used as the sole source of nutrition for infants or in combination with some breastfeeding. Decades of research and innovation have resulted in infant formulas with a more similar composition to human milk, yet there remain compositional differences between the two [2]. Thus, ongoing research in the area of pediatric nutrition seeks to identify components of human milk that may confer physiological benefits to the neonate and may be added to the infant formula matrix. One such class of dietary components that currently differ in concentration between human milk and infant formula are oligosaccharides (OS).

As the third most abundant component of human milk, OS are thought to aid in gastrointestinal development, brain development and prevention of pathogenic events [3]. Known differences in OS concentration and composition exist between human milk and milk from other mammalian species. Notably, mature human milk contains between 3.5–14 g OS/L, whereas mature bovine milk, which is often used as a base for infant formula, contains only 0.3–0.5 g OS/L and infant formulas are reported to contain 0.4–8.0 g OS/L [4]. Additionally, 50–70% of human milk OS are fucosylated (i.e., containing a fucose molecule, neutral OS), followed by 10–30% sialylated human milk OS (i.e., containing a sialic acid (SA) molecule, acidic OS) and approximately 10% of human milk OS are neutral OS that do not contain either a fucose or SA [5].

Among the acidic OS, the most abundant human milk OS is sialyllactose (SL), which is a trisaccharide molecule composed of a SA molecule bound to lactose. Sialyllactose in milk is found predominantly in two forms, 3′-SL and 6′-SL, with the number denoting the position of the SA monosaccharide linkage to lactose [6]. Interestingly, SL has been found in many mammalian milks, including human, bovine, murine and porcine, however the concentrations of total SL and the predominant isoform of SL (i.e., either 3′-SL or 6′-SL) varies widely among species [4,7,8]. Sialyllactose contains SA which may influence neonatal brain development. Sialic acid can be obtained from the diet or produced de novo and is incorporated into glycolipid- and glycoprotein-containing molecules. Notably, SA-containing glycolipids and glycoproteins are important for synapse formation and neural transmission [9], supporting the potential importance of this molecule during a highly dynamic period of brain development. In fact, breastfed infants exhibit increased brain SA concentrations relative to formula-fed infants, further suggesting the need for SA early in life [10]. Much like the wide variation of SL concentrations in mammalian milks, brain SA concentrations appear to vary widely among species, with humans exhibiting the highest concentrations among higher order mammals [11].

Early in life, the brain is highly dynamic and thus sensitive to dietary intervention. Provided the increasing interest in milk OS and emerging evidence suggesting dietary SA may influence the brain, there exists a need to identify clinically-relevant doses at which SA-containing milk OS might support neurodevelopment. The pig is an ideal translational model for assessing the effects of nutrition on brain development, because of its similar nutritional requirements, comparable gastrointestinal development and strikingly similar brain growth trajectories [12]. Thus, the aim of this exploratory study was to elucidate the effects of varying doses of dietary SL on pig brain development using magnetic resonance imaging (MRI) and tissue SA quantification. In doing so, this study expands on current literature, which suggests the presence of SA in the diet supports early-life brain development.

## 2. Methods

### 2.1. Animal Care & Housing

Beginning at 2 days of age, 38 vaginally-derived intact male pigs were randomly assigned to one of four milk replacer diets, described below, until 32 or 33 days of age. The study was completed in 4 replicates (5–12 pigs per replicate), with pigs selected from a total of 14 litters, such that potential confounding effects of litter of origin and initial bodyweight were taken into account when allotting pigs to dietary treatments. A total of 8–11 pigs were included in each dietary treatment group. A total of 12 pigs per dietary treatment were initially started on the study, however several pigs were removed from the study for health reasons unrelated to dietary treatments. All pigs were housed in custom pig rearing units (87.6 cm length × 88.9 cm width × 50.8 cm height) fabricated with clear acrylic and stainless steel walls and vinyl-coated metal flooring. Caging units permitted pigs to see, hear, smell but not touch neighboring pigs. All pigs were provided a toy for enrichment and were allowed to physically interact with other pigs during daily cleaning (approximately 15 min per day). Pig rearing environment was maintained on a 12 h light/dark cycle, with light from 0600 to 1800 h and minimal light during the overnight dark phase. All animal care and experimental procedures were in accordance with National Research Council Guide for the Care and Use of Laboratory Animals and approved by the University of Illinois at Urbana-Champaign Institutional Animal Care and Use Committee. Approval for this research project was verified on 3 March 2015 and is identified as IACUC 15034 at the University of Illinois Urbana-Champaign.

### 2.2. Dietary Treatments

All diets were produced by Mead Johnson Nutrition (Evansville, IN, USA) using a proprietary blend of nutrients formulated to meet the nutritional needs of growing pigs. Pigs were provided one of 4 custom diets from 2 until 32 or 33 days of age. The control diet (CON) included docosahexaenoic acid (DHA, 87 mg/100 g milk replacer powder; DSM, Heerlen, The Netherlands), arachidonic acid (ARA, 174 mg/100 g milk replacer powder; DSM, Heerlen, The Netherlands), galactooligosaccharide (GOS, 1.0 g/100 g milk replacer powder; Friesland Campina, Zwolle, The Netherlands) and polydextrose (PDX, 1.0 g/100 g milk replacer powder; Danisco, Terre Haute, IN, USA). Experimental diets were formulated using the CON diet as the base and supplemented with bovine-derived modified whey enriched with SL (SAL-10; Arla Foods Ingredients, Aarhus, Denmark) to provide final SL concentrations of: 65 mg SL/100 g milk replacer powder (LOW), 190 mg SL/100 g milk replacer powder (MOD) and 380 mg SL/100 g milk replacer powder (HIGH). The CON diet was composed of 30% protein, 32% fat, 29% carbohydrate, 8% ash and 1% water, all test diets (i.e., LOW, MOD, HIGH) were composed of 31% protein, 32% fat, 28% carbohydrate, 8% ash and 1% water.

Milk replacer powder was reconstituted fresh each day at 200 g of dry powder per 800 g of water and pigs were fed at 285 and 325 mL of reconstituted diet per kilogram of bodyweight (BW) starting on 3 and 8 days of age, respectively. At this reconstitution rate, all diets provided equal concentrations of DHA (174 mg/L), ARA (348 mg/L) and PDX/GOS (2 g/L, each). The reconstituted experimental milk replacers were formulated to contain 130 mg SL/L (LOW), 380 mg SL/L (MOD) or 760 mg SL/L (HIGH). However, analytical assessment conducted after study completion showed the levels of SL in the diets were: 55 mg SL/L (CON), 159 mg SL/L (LOW), 429 mg SL/L (MOD) and 779 mg SL/L (HIGH) due to inherent SL in the CON diet.

### 2.3. Sialic Acid Quantification

For quantification of free SA, serum and tissue samples from the right hemisphere hippocampus, cerebellum and prefrontal cortex were utilized. For brain tissue, all samples were homogenized using a bead homogenization system (TissueLyser, Qiagen, Hilgden, Germany) with 3 parts of phosphate buffered saline added to 1 part of brain tissue. Serum and brain homogenate were diluted 1:10 with nanopure water, homogenized for 1 h, further diluted 1:4 with 0.10 M sodium acetate solution (brought to a pH of 5 with HCl; Fischer Scientific, Hampton, NH, USA) and filtered into an auto sampler vial for analysis. The final dilution of the free SA samples was 1:40. Acetonitrile (Sigma-Aldrich, St. Louis, MO, USA) was used in both the standards and the samples to clarify the solutions. For quantification of total SA, the samples were diluted 1:25 with nanopure water and homogenized for 1 h. Next, 250 µL of diluted sample was combined with 750 µL of enzyme solution (neuraminidase (Roche Diagnostics, Indianapolis, IN, USA) and sodium acetate buffer) and digested for 18 h in a 37 °C water bath. Post digestion, the samples were filtered directly into an auto sampler vial for analysis. The final dilution of the total SA samples was 1:100. Again, acetonitrile was used in both the standards and the samples to clarify the solutions. For analysis of both the free and total SA, samples were analyzed by ion chromatography using pulsed amperometric detection (model ICS-5000, Dionex, Sunnyvale, CA, USA). Sialic acid separation was achieved using a CarboPac PA20 column (Dionex, Sunnyvale, CA, USA) and a multi-step gradient of 0–95 mM sodium acetate in 100 mM sodium hydroxide (Fischer Scientific, Hampton, NH, USA). Sialic acid concentrations were determined via an external standard calibration curve. *N*-acetylneuramic acid (Neu5Ac standard) and *N*-glycolylneuraminic acid (Neu5Gc standard) standards were used (Sigma-Aldrich, St. Louis, MO, USA). Concentrations of free and total SA were generated from the procedures described above and concentrations of bound SA were determined by subtracting free SA from total SA concentrations, within subject. Only concentrations for Neu5Ac were above detectable limits, thus no analysis of Neu5Gc concentrations are reported herein. The ratio of free SA to bound SA was determined by dividing free SA concentrations by bound SA concentrations, within subject.

### 2.4. Magnetic Resonance Imaging

All pigs underwent MRI procedures on postnatal day 32 or 33 at the Beckman Institute Biomedical Imaging Center at the University of Illinois using a Siemens MAGNETOM Trio 3T scanner (Siemens Healthineers, Erlangen, Germany) with a Siemens 32-channel head coil. The pig neuroimaging protocol included three magnetization prepared rapid gradient-echo (MPRAGE) sequences and diffusion tensor imaging (DTI) to assess brain macrostructure and microstructure, respectively, as well as magnetic resonance spectroscopy (MRS) to obtain brain metabolite concentrations [13]. In preparation for MRI procedures, anesthesia was induced using an intramuscular injection of telazol (50.0 mg of tiletamine plus 50.0 mg of zolazepam reconstituted with 5.0 mL deionized water; Zoetis, Florham Park, NJ, USA) administered at 0.07 mL/kg BW and an appropriate plane of anesthesia was maintained with inhaled isoflurane (98% O_2_, 2% isoflurane) delivered via a mask. Pigs were immobilized during all MRI procedures. Visual observation of each pig’s well-being, as well as observations of heart rate, PO_2_ and percent of isoflurane, were recorded every 5 min during the procedure and every 10 min post-procedure until animals were fully recovered. Total scan time for each pig was approximately 60 min.

#### 2.4.1. Diffusion Tensor Imaging Acquisition and Analysis

Diffusion tensor imaging was used to assess white matter maturation and axonal tract integrity using a *b*-value = 1000 s/mm^2^ across 30 directions and a 2 mm isotropic voxel. Diffusion-weighted echo-planar imaging (EPI) images were assessed using FMRIB Software Library (FSL) (FMRIB Centre, Oxford, UK) for fractional anisotropy (FA), mean diffusivity (MD), axial diffusivity (AD) and radial diffusivity (RD) using methods previously described [13]. Assessment was performed over the following regions of interest: caudate, corpus callosum, cerebellum, both hippocampi, internal capsule, left and right cortex, thalamus, DTI-generated white matter and atlas-generated white matter was performed using a customized pig analysis pipeline and the FSL software package. For the purposes of this analysis this study used the Pig Brain atlas, generated from the same species and previously reported by Conrad and colleagues [14]. The diffusion toolbox in FSL was used to generate values of AD, RD, MD and FA.

Masks for each region of interest (ROI) from the atlas were non-linearly transformed into the MPRAGE space of each individual pig and a linear transform was then applied to transfer each ROI into DTI space. A threshold of 0.5 was applied to each ROI and the data were dilated twice. For each individual ROI, an FA threshold of 0.15 was applied to ensure inclusion of only white matter in the region of interest despite the mask expansion.

#### 2.4.2. Tract-Based Spatial Statistics

The FSL 5.0 toolbox was used for tract-based spatial statistics (TBSS) assessment of FA data. Fractional anisotropy images, previously generated from diffusion data, were manually extracted and all FA data from individual subjects were aligned using the FSL nonlinear registration tool FNIRT. Upon alignment, the study-specific mean FA image was created and a mean FA skeleton representing the center of all common tracts was established. A threshold of 0.2 was determined to be sensitive for mean FA tract delineation. Once the study-specific mean FA skeleton was created, each subjects’ aligned FA data were projected onto the mean FA skeleton and the resulting voxel-wise cross-subject data were used for statistical analyses. For TBSS analysis, only pigs receiving the LOW and MOD diets were compared, thus a two-sample *t*-test was used for data analysis. Comparison between only the LOW and MOD diets was performed prior to un-blinding of dietary treatment. These treatments were specifically selected for comparison analysis based on the results of the DTI measurements. In particular, the difference between LOW and MOD in DTI analysis was the greatest of all comparisons.

The TBSS non-FA function was used to generate data on diffusion differences along the pre-determined white matter tracts for other diffusion tensor measures (i.e., MD, AD, RD). To analyze differences in these diffusion measures, nonlinear warps and skeleton projection values generated in the TBSS FA analysis were applied to each of the MD, AD and RD images. Pig-specific alterations to the non-FA script include registration to pig brain atlas space, rather than MNI152 space and registration using a pig-specific internal capsule mask rather than a lower cingulum mask. Statistical analysis for each of these diffusion measures was performed as described above for FA analysis.

#### 2.4.3. Structural MRI Acquisition and Analysis

A T1-weighted MPRAGE sequence was used to obtain anatomic images of the pig brain, with a 0.7 isotropic voxel size. Three repetitions were acquired and averaged using SPM8 in Matlab 8.3 and brains were manually extracted using FSL (FMRIB Centre, Oxford, UK). The following sequence specific parameters were used to acquire T1-weighted MPRAGE data: repetition time (TR) = 1900 ms; echo time (TE) = 2.49 ms; 224 slices; field of view (FOV) = 180 mm; flip angle = 9°. Methods for MPRAGE averaging, manual brain extraction were previously described [13]. All data generated used a publicly-available population-averaged pig brain atlas (http://pigmri.illinois.edu) [14].

For volumetric assessments, individual brains were segmented into 19 different ROI using the pig brain atlas. Total brain and individual region volume analysis was performed in which an inverse warp file for each ROI was generated from the DARTEL-generated warp files for each region using the using the statistical parametric mapping (SPM8; Wellcome Department of Clinical Neurology, London, UK) software. Generation of region-specific warp files was previously described [15,16]. In order to account absolute whole-brain volume, all regions of interest were also expressed as a percent of total brain volume (%TBV), using the following equation: ((ROI absolute volume)/(total brain absolute volume)) × 100, within subject.

Voxel-based morphometry (VBM) analysis was performed to assess grey and white matter tissue concentrations using SPM8 software (Wellcome Department of Clinical Neurology, London, UK). Manually-extracted brains were aligned to pig brain atlas space using a 12-parameter affine transformation. The “Segment” function of SPM and pig-specific prior probability tissue maps were then used to segment the brains into grey matter and white matter. The Diffeomorphic Anatomical Registration using Exponentiated Lie Algebra (DARTEL) toolbox was used with pig-specific specifications that included changing the bounding box of −30.1 to 30.1, −35 to 44.8, −28 to 31.5; and a voxel size of 0.7 mm^3^. After the nonlinear transformation of the data in the DARTEL procedure, flow fields were created and converted to warp files. The warp files generated were then applied to the subject’s grey and white matter. The modulated data were smoothed with a 4 mm full-width half maximum (FWHM) and were subjected to VBM procedures using the SPM8 toolbox. For voxel-based morphometry analyses, two-sample permutation *t*-tests were performed on a voxel-by-voxel basis for grey and white matter volume differences between animals on the LOW and MOD diets, with an uncorrected *p <* 0.001. An additional threshold criterion of at least 20-edge connected voxels was used. Comparison between only the LOW and MOD diets was performed prior to un-blinding of dietary treatment. These treatments were chosen for comparison based off of the greatest difference in diffusion tensor imaging values between the treatments.

#### 2.4.4. Magnetic Resonance Spectroscopy Acquisition and Analysis

Magnetic resonance spectroscopy was used to non-invasively quantify metabolites in the whole brain. The MRS spin-echo chemical shift sequence was used with a voxel size of 20 mm × 23 mm × 13 mm and centered over the left and right dorsal hippocampi. The following sequence parameters were used in acquisition of spectroscopy data for the water suppressed scan TR = 1800 ms; TE = 68 ms; signal averages = 256, vector size = 1024. The following sequence parameters were used in acquisition of spectroscopy data for the non-water suppressed scan TR = 20,000 ms; TE = 68 ms; signal averages = 1; vector size = 1024 point. Both water-suppressed and non-water-suppressed data were collected in institutional units and all MRS data were analyzed using LC Model (version 6.3), using methods previously described [15]. Limits were placed on MRS data for inclusion in the statistical analysis. Cramer-Rao lower bounds (i.e., % standard deviation) were calculated using the LC Model and only metabolites with standard deviation less than 20% were considered to have reliable quantitative results of absolute levels.

### 2.5. Statistical Analysis

All researchers involved with conducting the study and acquiring and analyzing the study results remained blinded to dietary treatment identity until final data analyses had been completed. An analysis of variance (ANOVA) was conducted using the MIXED procedure of SAS 9.4 (SAS Inst. Inc., Cary, NC, USA) to differentiate the effects of the dietary treatments provided to the pigs. All data analyzed herein were collected at a single time-point and were thus analyzed using a one-way ANOVA. No outliers (i.e., individual data-points greater than |3| studentized residuals away from the treatment mean) were detected for any measurements. The level of significance was set at *p <* 0.05 with trends accepted at 0.05 < *p <* 0.10.

## 3. Results

### 3.1. Tissue & Serum Sialic Acid Quantification

**Figure 1:**
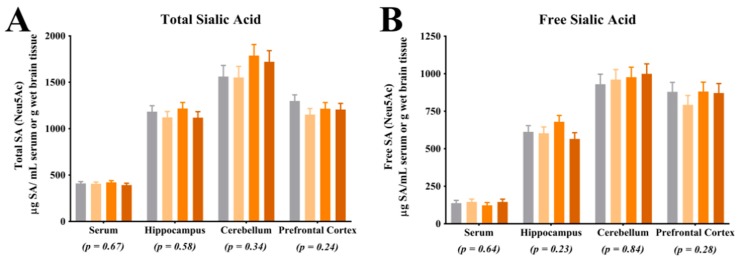

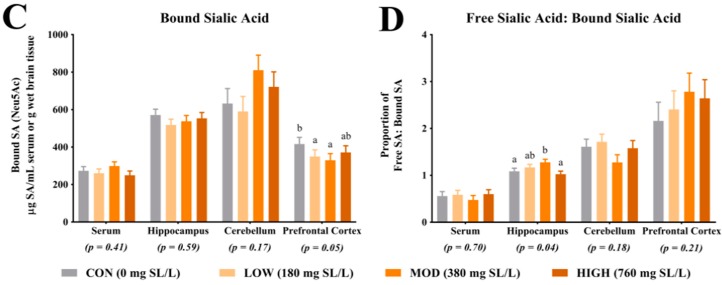
Bound sialic acid (SA) in the prefrontal cortex and the ratio of free SA to bound SA in the hippocampus are influenced by dietary sialyllactose (SL). (**A**) Dietary SL does not influence (*p* > 0.05) total SA in serum, hippocampus, cerebellum and prefrontal cortex; (**B**) Dietary SL does not influence (*p* > 0.05) free SA in serum, hippocampus, cerebellum and prefrontal cortex; (**C**) Dietary SL influences (*p* = 0.05) the concentration of bound SA in the prefrontal cortex; (**D**) Dietary SL influences (*p* = 0.04) the ratio of free SA to bound SA in the hippocampus. Note, serum SA concentrations are in µg/mL serum whereas brain tissue SA concentrations are in µg/g brain tissue.

For all SA quantification measures, the following number of pigs were analyzed per dietary treatment: CON (*n* = 11), LOW (*n* = 9), MOD (*n* = 10), HIGH (*n* = 8). Analysis of total and free SA indicated no differences between dietary treatments in the serum (*p* = 0.67 and 0.64, respectively), hippocampus (*p* = 0.58 and 0.23, respectively), cerebellum (*p* = 0.34 and 0.84, respectively) and prefrontal cortex (*p* = 0.24 and 0.28, respectively) (Figure 1A,B). Analysis of bound SA indicated an effect of dietary treatment (*p* = 0.05) in the prefrontal cortex. Within the prefrontal cortex, pigs on the CON diet group exhibited higher levels of bound SA compared with the LOW and MOD dietary SL groups but were not different than the HIGH dietary SL group (Figure 1C). Analysis of bound SA indicated no differences between dietary treatments in the serum (*p* = 0.41), hippocampus (*p* = 0.59), or cerebellum (*p* = 0.17). Analysis of free SA:bound SA indicated an effect of diet (*p* = 0.04) in the hippocampus, in which the MOD group exhibited a higher proportion of free SA:bound SA compared with the CON and HIGH diet groups but was not different than the LOW group (Figure 1D). Analysis of free SA:bound SA indicated no differences between dietary treatments in the serum (*p* = 0.70), cerebellum (*p* = 0.18) and prefrontal cortex (*p* = 0.21).

### 3.2. Magnetic Resonance Imaging

#### 3.2.1. Diffusion Tensor Imaging

**Figure 2:**
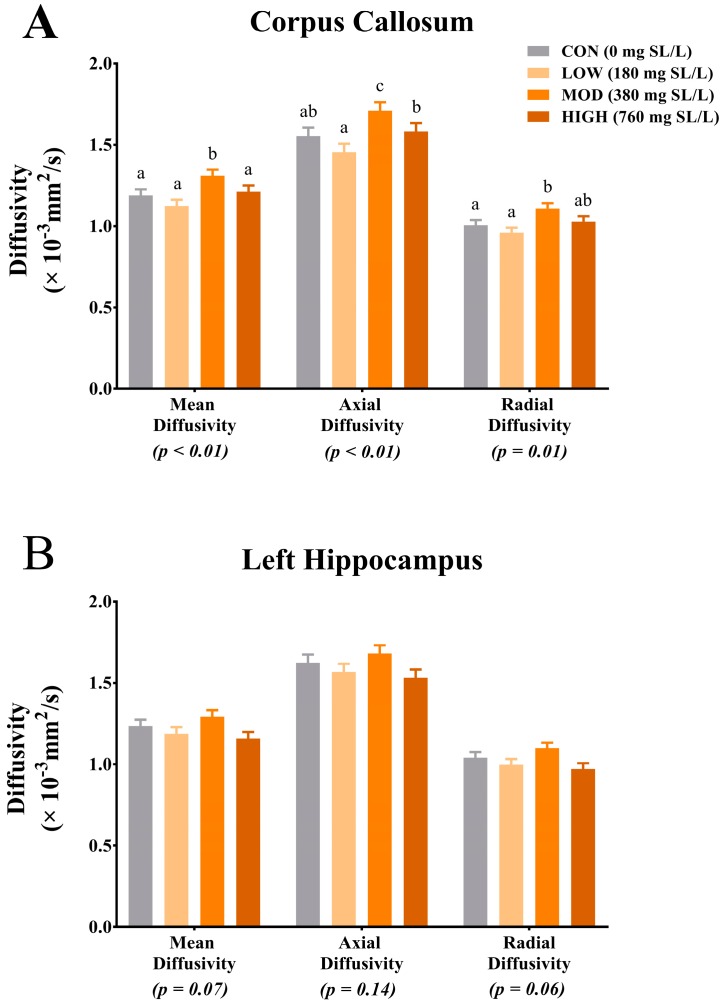
Diffusion tensor measures in the corpus callosum are influenced by dietary sialyllactose (SL). (**A**) Dietary SL influences diffusion tensor measures of mean diffusivity (MD) (*p <* 0.01), axial diffusivity (*p <* 0.01) and radial diffusivity (RD) (*p* = 0.01) in the corpus callosum. For all measures, pigs provided the MOD dietary SL treatment exhibited the highest rates of diffusion; (**B**) Dietary SL tended to influence (0.05 < *p <* 0.10) diffusion tensor measures of MD (*p* = 0.07) and RD (*p* = 0.06) in the left hippocampus.

Due to motion artifact in some of the acquired scans the following number of pigs were analyzed per dietary treatment: CON (*n* = 9), LOW (*n* = 9), MOD (*n* = 7), HIGH (*n* = 7). Diffusion tensor imaging revealed differences due to diet in corpus callosum AD (*p <* 0.01), MD (*p <* 0.01) and RD (*p* = 0.01) measures (Figure 2A). Corpus callosum MD measures were highest in the MOD group compared with CON, LOW and HIGH, which were not different from each other. Axial diffusivity measures in the corpus callosum indicated highest rates of diffusion in the MOD group when compared with all dietary treatments and HIGH pigs exhibited increased rates of diffusivity compared with LOW but not CON pigs. For RD measures, rates of diffusion were highest in MOD pigs but not different than HIGH pigs, however, rates of RD in the HIGH pigs were also not different than CON and LOW pigs. Trends for main effect of diet were observed for left hippocampus MD (*p* = 0.07) and RD (*p* = 0.06) measures, with the MOD pigs exhibiting numerically higher but not statistically significant, rates of diffusion compared with all other groups (Figure 2B). No differences due to diet were observed for MD, AD and RD measures in other analyzed ROI. Additionally, there were no differences in fractional anisotropy measures in any of the analyzed brain regions (data not shown).

#### 3.2.2. Tract-Based Spatial Statistics

**Figure 3:**
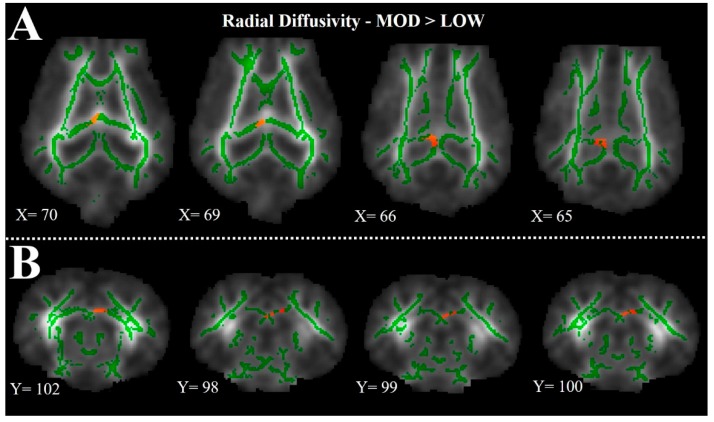
Tract-based spatial statistics (TBSS) illustrating differences in radial diffusivity (RD) between LOW and MOD dietary sialyllactose (SL) pigs. Pigs provided the MOD dietary SL treatment exhibited higher (*p* < 0.05) rates of radial diffusivity in the left hemisphere corpus callosum, when compared with LOW dietary SL pigs. The images generated from TBSS are and average of all LOW and MOD dietary SL pigs, green lines indicate regions in which all pigs exhibited white matter voxels. Representative slices were chosen to highlight areas in which RD values in MOD dietary SL pigs were significantly (*p* < 0.05) different compared with LOW dietary SL pigs. (**A**) Axial slices, with varying X-coordinates and static Y = 120 and Z = 76 coordinates, determined using University of Illinois Pig Brain Atlas [14]; (**B**) Coronal slices, with varying Y-coordinates and static X = 73 and Z = 87 coordinates, determined using the University of Illinois Pig Brain Atlas. Dark red and light red colors indicate degree of statistical differences from *p* = 0.05 to *p* < 0.01, respectively. For a more comprehensive image set of TBSS images the reader is referred to Supplemental Figure S1.

Tract-based spatial statistics (TBSS) analysis was used to identify diffusion differences between LOW diet and MOD diet pigs using study-specific, pre-determined white matter tracts. TBSS analysis of RD measures indicated a cluster of voxels in which MOD pigs exhibited higher (*p <* 0.05) RD measures when compared with LOW pigs (Figure 3, Figure S1 for full image set). Upon visual inspection, the significant voxels appear to be localized to the left hemisphere corpus callosum. The converse of this voxel-wise comparison yielded no differences in RD measures, indicating LOW pigs did not exhibit higher RD measures compared with MOD pigs, which supports DTI findings in the corpus callosum.

Tract-based spatial statistics analyses were also performed on skeletonized masks of FA, AD and MD data. For each separate analysis, diffusion values revealed no differences (*p >* 0.05) in which pigs provided the LOW diet exhibited higher diffusion values along predetermined white matter tracts, when compared with pigs fed the MOD diet. Further analysis revealed no differences (*p >* 0.05) along the same predetermined white matter tracts in which pigs provided MOD diet exhibited higher FA, AD, or MD values when compared with pigs provided LOW diet.

#### 3.2.3. Voxel-Based Morphometry

**Figure 4:**
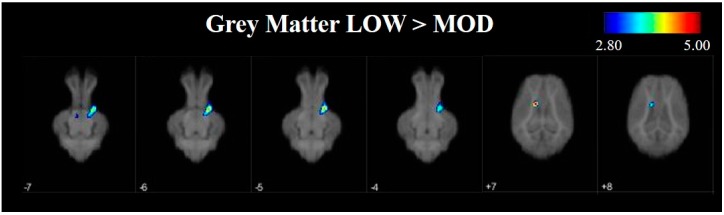
Voxel-based morphometry (VBM) heat maps illustrating grey matter tissue concentration differences between LOW and MOD dietary sialyllactose (SL) pigs. The colored bar indicates pseudo-t statistics, used to determine the *p*-uncorrected statistics provided in Table 1. Shown above are areas in which LOW dietary SL pigs exhibited greater (*p* < 0.01) concentrations of grey matter when compared with MOD dietary SL pigs. For a more comprehensive set of VBM images the reader is referred to Supplemental Figure S2.

Voxel-based morphometry analysis identified differences in the location and size of tissue clusters between LOW and MOD dietary SL groups. Grey matter analysis revealed the largest and most significant cluster differences in which LOW pigs exhibited greater (*p <* 0.05) concentrations of grey matter when compared with MOD SL pigs (Table 1, Figure 4 and Figure S2 for full image set). These observed differences in which LOW > MOD were primarily localized to cortical tissue. Additionally, significant clusters revealed differences in cortical grey matter where MOD pigs exhibited larger concentrations when compared with LOW pigs; however, these clusters generally contained fewer voxels than the opposite comparison (i.e., MOD > LOW).

Differences between LOW and MOD pig white matter concentrations were also evident, although these clusters contained fewer voxels than those observed in grey matter comparisons. Pigs provided the LOW diet exhibited higher (*p <* 0.05) concentrations of white matter in subcortical brain regions (i.e., caudate and midbrain) when compared with MOD pigs. Analysis of the reverse comparison (i.e., MOD > LOW) revealed that MOD pigs showed greater (*p <* 0.05) white matter concentrations in the pons and left cortex, compared with LOW pigs.

#### 3.2.4. Brain Volumes

Due to excessive motion artifact in some of the acquired scans, the following number of pigs were analyzed per dietary treatment: CON (*n* = 10), LOW (*n* = 9), MOD (*n* = 9), HIGH (*n* = 8). No differences between absolute total brain volumes were observed between dietary treatments. Analysis of absolute volume in 19 different ROI did not result in differences due to diet (Table S1). Relative brain volume measures were generated by dividing an individual region of interest by the total brain volume, within subject and data is presented as a percent of total brain volume. No dietary effects were noted for relative brain volumes (Table S2). A trend (*p* = 0.06) for relative size of the left cortex was observed.

#### 3.2.5. Single-Voxel Spectroscopy

Due to excessive motion artifact in some of the acquired scans the following number of pigs were analyzed per dietary treatment: CON (*n* = 10), LOW (*n* = 9), MOD (*n* = 7), HIGH (*n* = 7). Single-voxel spectroscopy revealed no difference due to diet in any of the seven measured metabolites. A trend for main effect of diet (*p* = 0.07) was observed for glutamate, with MOD pigs exhibiting numerically lower but not statistically significant, concentrations of glutamate when compared with all other groups (Table S3).

## 4. Discussion

Human milk OS are the third most abundant component in human milk and appear to be pivotal for proper neonatal development [4]. Approximately 10–30% of human milk OS are SA-containing, acidic OS, predominantly 3′-SL and 6′-SL [5,6]. Based on the high concentration of SL in human milk and the known roles of SA in brain development, there is interest in determining how dietary SL might influence neurodevelopment. This study assesses the effects of dietary SL on pig brain development using MRI and supports previous work assessing the effects of dietary SL on brain development in the biomedical pig model [17]. Results from this study indicate physiologically relevant levels of dietary SL influence SA tissue concentrations in the prefrontal cortex and hippocampus of young pigs, DTI measures in the corpus callosum and cortical grey and white matter tissue concentrations.

Supplementation of dietary compounds that contain SA have previously been reported to influence brain development but the ingredient used to supply SA varies widely. For example, dietary supplementation of casein glycomacropeptide, a SA-containing compound that is not a pure OS, increased learning and memory, raised cortical protein-bound SA concentrations and influenced the expression of learning-related genes in the five-week-old pig [18]. Moreover, intravenous infusion of C^14^-labeled SA confirmed the ability of SA to cross the blood-brain-barrier and showed preferential accretion of labeled SA in the cortex of three-day-old pigs [19]. Provision of dietary SL has been shown to influence total SA and ganglioside-bound SA concentrations in the corpus callosum and cerebellum of three-week-old pigs [17]. Moreover, rats that were provided SA in the form of SL or galactosylated SA exhibited improved learning and increased brain SA and ganglioside concentrations compared with control rats [20]. A recent study suggested decreased stressor-induced anxiety-like behaviors in mice provided dietary SL compared with control mice [21]. These findings indicate an overt influence of dietary SA-containing compounds on the developing brain, however the optimal levels of supplementation and specific physiological effects of each compound remain to be elucidated.

Our study supplemented a modified whey protein enriched with a mixture of 3′-SL and 6′-SL at concentrations similar to mature sow milk (CON), mature bovine milk (LOW), 61-120 day human milk (MOD) and double what is typically found in mature human milk (HIGH) [4]. Notably, the LOW, MOD and HIGH dietary SL concentrations were formulated to be above the published concentrations of SL in infant formulas [4]. Analytical assessment conducted of the final diets indicated levels of SL as follows: CON (55 mg SL/L), LOW (159 mg SL/L), MOD (429 mg/L) and HIGH (779 mg/L). These concentrations are likely due to inherent SL from bovine milk-derived ingredient sources used in the CON diet. The supplementation levels in the present study are lower compared with a recent pig study, which fed formula supplemented with pure forms of 3′-SL or 6′-SL at either 2 g/L or 4 g/L [17], which are greater than the SL concentrations of human colostrum [4]. To our knowledge, the study by Jacobi and colleagues and the present study are the only studies to assess the influence of dietary SL on brain development using the biomedical pig model.

### 4.1. Sialic Acid Concentrations

Analysis of SA in the prefrontal cortex indicated a greater proportion of bound SA in CON pigs when compared with LOW and MOD dietary SL pigs. Interestingly, concentrations of bound SA were not different between CON and HIGH dietary SL pigs. This study did not differentiate between SA bound to gangliosides or glycoproteins and it is unclear if the reduced concentration of bound SA in the LOW and MOD dietary SL groups is of physiological relevance. Results from Wang and colleagues [18] indicated slight increases in frontal cortex protein-bound SA when cGMP concentrations were 300, 635 and 830 mg/L compared with a 140 mg/L control and no differences in ganglioside-bound SA were noted between any treatment groups. When comparing our results (SL supplementation) with those from the study by Wang and colleagues (cGMP supplementation), it appears that structural specificity of dietary compounds that carry SA may be influential in SA accretion in the brain.

Herein, the ratio of free-to-bound SA indicated a higher proportion in the hippocampus of MOD pigs compared with CON and HIGH pigs but no difference between MOD and LOW pigs. Brain development is a heterogeneous process with brain regions developing at different rates [22]. It is known that the hippocampus is rapidly developing at 4-weeks of age in the pig [23] and research in chimpanzees indicates SA concentrations increase in the brain throughout development [11]. A study that supplemented three-day-old pigs with isotopically-labeled SA showed differences in brain region SA accretion after labeled SA injection, further suggesting region-specific accretion of SA during a highly dynamic period of neurodevelopment [19]. Wang and colleagues further suggest that the rate of SA accretion may be dependent on the species, subject age, dietary form of SA, and route of administration. Therefore, based on evidence from our study, we speculate that this increase in free SA relative to bound SA in the hippocampus could indicate a highly metabolic brain region that may be preparing SA to be incorporated into other compounds (e.g., glycoproteins or glycolipids) by preferentially partitioning free SA at the time of analysis. It is known that ganglioside concentrations in the brain rapidly increase perinatally [24], thus, future work should seek to characterize changes in free SA relative to bound SA in various brain regions over time to better understand the importance of this observation.

Assessment of total and free SA indicated no differences in their concentrations in the prefrontal cortex, cerebellum, or hippocampus. The lack of difference in total SA and free SA in these regions is in agreement with observations in a previous pig study, where researchers also did not observe differences in these regions in pigs that were provided pure forms of dietary SL [17]. While interesting, caution should be exercised when making comparisons between these two studies as the concentrations of dietary SL differed markedly, as described above. Notably, the lack of difference in blood SA concentrations may be explained through several different mechanisms. First, it is possible that circulating SA concentrations are tightly regulated in the body and endogenous synthesis of SA could have compensated for the difference in dietary SA. Additionally, it is possible that there is a limit at which dietary SA is bioavailable and the limit may have been met in each dietary treatment. Blood was only collected at the end of the study and it is also possible that differences in circulating SA concentrations existed earlier in the study but were corrected through compensatory mechanisms by the end of the study. Lastly, SL may have acted on gut microbiota and the microbiota may have influenced other circulating compounds which affected brain development. Future work should seek to quantify circulating SA concentrations at multiple time points, characterize bioavailability of dietary SA and assess the influence of the microbiome-gut-brain-axis.

### 4.2. Magnetic Resonance Imaging

Diffusion tensor imaging permits characterization of tissue microstructure through assessment of microscopic water movement in the brain [25]. Results from our study indicate differences due to dietary treatment in MD, AD and RD within the corpus callosum. Interestingly, for each measure, pigs that were provided MOD dietary SL exhibited the highest rates of diffusion across all diffusion measures. Moreover, for each diffusion measure (i.e., MD, AD and RD), pigs provided the HIGH dietary SL treatment exhibited reduced diffusivity when compared with the MOD dietary SL pigs. Provided the MOD dietary SL treatment was formulated to mimic mature human milk concentrations, it is possible that the HIGH dietary SL level (i.e., twice what is commonly observed in human milk) may not confer any added benefit to development. Although our pig study was not designed to look at specific morphology of brain structures, it is possible that the differences in diffusion properties between MOD and all other dietary treatments are a result of differences in corpus callosum structural architecture. A study of diffusion measures in the adult human corpus callosum indicates differences in fractional anisotropy among various sections of the corpus callosum [26]. These differences in diffusion values in the corpus callosum segments correspond to known structural differences (i.e., axon diameter, axon myelination and fiber packing) in each segment. Thus, future work should seek to characterize axon diameter, myelin thickness and density of fiber packing in the corpus callosum after a dietary SL intervention. While only trending, MD and RD in the left hippocampus indicated numerically higher rates of diffusion in the MOD dietary SL pigs compared with the CON, LOW and HIGH pigs. Interestingly, these observed trends for hippocampal MD and RD follow the same pattern as what was observed for free SA relative to bound SA concentrations in the hippocampus but it is unclear if the two are related. Also of note, the diffusion values observed across treatment groups in this study appear to be within the range of what has previously been reported and thus do not indicate any safety concerns.

The apparent sensitivity of the corpus callosum to dietary SL again support findings from a recent pig study, which showed increased ganglioside SA accretion in the corpus callosum of pigs supplemented with either 2 g/L 3′-SL or 6′-SL, compared with pigs receiving 4 g/L of 3′-SL or 6′-SL [17]. Despite the stark differences in dietary SL concentrations between the two studies, it is interesting to note that neither study observed linear increases in outcomes measures with higher supplementation rates. Jacobi and colleagues observed a quadratic effect for SL supplementation with the middle supplementation rate (i.e., 2 g SL/L compared with 0 g SL/L and 4 g SL/L) conferring the greatest total SA and ganglioside-bound SA accretion in the corpus callosum. Although we did not statistically test quadratic effects, anecdotally, similar patterns were observed with the MOD dietary SL group exhibiting the highest rates of diffusion relative to CON, LOW and HIGH dietary SL pigs. Together these findings may suggest an optimal window at which dietary SL modulates corpus callosum development.

To better characterize the observations of corpus callosum DTI findings, we also assessed the difference between the LOW and MOD dietary SL treatments using TBSS. Tract-based spatial statistics provides a visual comparison between treatment groups, thereby elucidating areas within white matter tracts where structural differences may exist. From the heat maps generated in this analysis, it is clear that the largest differences in RD of the corpus callosum exist in the left hemisphere. A study of two-year-old chimpanzee cortical brain tissue indicates greater SA accretion in the left hemisphere compared with the right hemisphere [11]. Although this study did not assess corpus callosum SA enrichment, these findings, coupled with the diffusion trends in the left hippocampus and left hemisphere corpus callosum TBSS observations, might suggest sensitivity of the left hemisphere to dietary forms of SA early in life. Also of note, the SA tissue quantification was performed on right hemisphere samples. Provided the results presented above and work by Wang and colleagues [11], it is possible that more robust dietary effects may have been observed in left hemisphere tissue analyses.

To further investigate differences in grey and white matter development, VBM was used to compare tissue concentrations between LOW and MOD dietary SL pigs. Pigs that were provided the LOW dietary SL treatment appeared to have greater concentrations of grey matter in the right cortex and the midbrain when compared with MOD dietary SL pigs. Conversely, assessment of pigs provided the MOD diet appeared to have areas of increased grey matter in localized areas of left cortex when compared with LOW dietary SL pigs. It is known that grey matter tissue concentrations change throughout development [27], however it is unclear if these changes may be due to axonal growth or synaptic pruning, each of which may influence grey matter tissue concentrations. Future work should seek to characterize markers of synaptic pruning and axon growth to better understand if decreased tissue concentrations in MOD dietary SL pigs may be a result of pruning or growth events.

### 4.3. Limitations

A recent study of porcine milk OS suggests 3′-SL is the predominant acidic OS with a concentration of 100 mg/L in colostrum that falls below 50 mg/L by day 7 of lactation [8]. Provided the low concentrations of 3′-SL and undetectable concentrations of 6′-SL in porcine milk, it is possible that all concentrations of dietary SL used in this study were sufficient to support normal brain development and future work should seek to compare with a control diet which does not contain endogenous SL. Additionally, it remains unclear exactly how SL is digested and absorbed into the body. The findings presented herein may be a result of dietary SL directly providing SA for brain development. However, recent evidence in the pig model suggests alterations in gut development may modulate particular aspects of brain development [28], therefore it is also possible that dietary SL may have stimulated gut development which in turn influenced brain development. In fact, dietary SL has been shown to alter proximal and distal colon microbiota in the pig [17], thus it is possible dietary SL may influence the brain through the gut-brain-axis. Future work should seek to characterize the metabolism of dietary SL and elucidate its main mode of influence in brain development. Also of note, a rat model of necrotizing enterocolitis showed a structure-dependent response to a specific SA-containing OS, thereby suggesting unique roles for each isomer of SA-containing OS [29]. This finding in the gut coupled with varying effects of SA-containing molecules in the brain suggest structure specific functions of SA molecules and their role in infant development. Therefore, future work should seek to sensitively characterize the unique role of individual dietary SA containing compounds, in order to elucidate their specific physiological relevance.

## 5. Conclusions

There is a lack of studies directly assessing the influence of dietary SL on brain development. The data from our study adds to a growing body of literature, which suggests brain development is sensitive to the presence of SL in the diet. To our knowledge, this is the first study to use magnetic resonance imaging to quantify differences in brain development due to dietary SL. Moreover, this study substantiates recent findings that the corpus callosum and left hemisphere appear to be influenced by the presence of SL in the diet.

## Figures and Tables

**Table 1:** Voxel-based morphometry comparison between pigs provided LOW and MOD dietary sialyllactose ^1^.

			Cluster	Peak Level		Local Maxima Coordinates ^3^		
Tissue	Comparison	Anatomic Region ^2^	(# Voxels)	*p*-Value	*Pseudo-t*	x	y	z
Grey	LOW > MOD	Lateral Ventricle/Corpus Callosum	141	<0.001	5.03	−4.9	14.0	7.0
		Right Cortex	1160	<0.001	4.5	9.1	10.5	−5.6
		Midbrain		0.001	4.02	−3.5	4.2	−9.8
		Midbrain/Right Cortex		0.007	2.8	7.7	2.8	−9.8
		Right Cortex	28	0.004	3.15	7.0	26.6	−2.8
		Left Cortex	163	0.004	3.13	−18.2	4.2	−2.1
	MOD > LOW	Left Cortex	139	<0.001	5.01	−14.7	4.2	11.9
		Right Cortex	39	0.001	3.74	12.6	0.7	11.9
		Left Cortex	23	0.004	3.1	−6.3	3.5	18.9
White	LOW > MOD	Caudate	128	0.001	3.95	2.1	18.9	2.8
		Midbrain/Right Cortex	93	0.001	3.74	7.7	3.5	−9.1
	MOD > LOW	Pons	147	<0.001	4.33	−6.3	−9.8	−9.1
		Left Cortex	32	0.007	2.85	−16.1	4.9	11.9
		Left Cortex	30	0.007	2.8	−12.6	0.7	12.6

^1^ Voxel-based morphometry analysis of grey and white matter differences in the LOW (*n* = 9) and MOD (*n* = 7) pig brains. A threshold of *p <* 0.01 and minimum cluster size of 20 voxels was used to determine uncorrected *p*-values listed in the table. ^2^ Brain regions based on estimates from the University of Illinois Pig Brain Atlas [14]. ^3^ Local Maxima Coordinates: X increases from left (−) to right (+), y increases from posterior (−) to anterior (+) and z increases from inferior (−) to superior (+). Abbreviations: # (number of voxels).

## Supplementary Materials

The following are available online at www.mdpi.com/2072-6643/9/12/1297/s1, Table S1: Dietary sialyllactose does not influence absolute brain volumes; Table S2: Dietary sialyllactose does not influence relative brain volumes; Table S3: Dietary sialyllactose does not affect single-voxel spectroscopy measures; Figure S1: LOW and MOD pigs exhibit differences in tract-based spatial statistics; Figure S2: LOW and MOD pigs exhibit differences in grey and white matter concentrations.
