# Supplementary material for: Dietary Sialyllactose Influences Sialic Acid Concentrations in the Prefrontal Cortex and Magnetic Resonance Imaging Measures in Corpus Callosum of Young Pigs
Source: Nutrients. 2017 Nov 28;9(12):1297. doi: 10.3390/nu9121297 (PMC5748748; doi:10.3390/nu9121297)
Supplement: Supplementary file 1 [file nutrients-09-01297-s001.pdf]

**Supplemental Table 1**. Dietary sialyllactose does not influence absolute brain volumes[1]

| *Region of Interest, ($mm^3$)* | **CON** | **LOW** | **MOD** | **HIGH** | **Pooled SEM** | ***p*-Value** |
|---|---|---|---|---|---|---|
| Whole Brain | 62785 | 61410 | 62287 | 60610 | 1693 | 0.76 |
| Grey Matter | 35428 | 34674 | 35886 | 34518 | 1146 | 0.76 |
| White Matter | 16684 | 16308 | 16441 | 16294 | 492 | 0.92 |
| Cerebrospinal Fluid | 5836 | 6659 | 6668 | 7111 | 668 | 0.39 |
| Caudate | 467 | 474 | 484 | 464 | 17.0 | 0.63 |
| Cerebellum | 5941 | 5796 | 6035 | 5903 | 187 | 0.75 |
| Cerebral Aqueduct | 43.1 | 46.4 | 44.8 | 44.8 | 1.60 | 0.38 |
| Corpus Callosum | 385 | 376 | 371 | 372 | 10.0 | 0.66 |
| Fourth Ventricle | 47.7 | 52.4 | 50.2 | 51.1 | 2.50 | 0.37 |
| Hypothalamus | 245 | 249 | 246 | 243 | 9.30 | 0.92 |
| Internal Capsule | 1513 | 1488 | 1487 | 1480 | 40.2 | 0.90 |
| Lateral Ventricle | 480 | 450 | 522 | 491 | 26.7 | 0.23 |
| Left Cortex | 16315 | 16394 | 16676 | 16224 | 445 | 0.88 |
| Left Hippocampus | 479 | 461 | 473 | 455 | 16.2 | 0.57 |
| Medulla | 1765 | 1724 | 1777 | 1788 | 53.7 | 0.79 |
| Midbrain | 1854 | 1826 | 1816 | 1814 | 50.2 | 0.84 |
| Olfactory Bulb | 2372 | 2357 | 2308 | 2287 | 66.4 | 0.75 |
| Pons | 1109 | 1074 | 1071 | 1077 | 28.3 | 0.40 |
| Putamen-Globus Pallidus | 405 | 387 | 400 | 389 | 11.5 | 0.44 |
| Right Cortex | 17031 | 16628 | 17257 | 16762 | 448.3 | 0.73 |
| Right Hippocampus | 489 | 474 | 481 | 466 | 15.7 | 0.62 |
| Thalamus | 1602 | 1573 | 1591 | 1563 | 45.0 | 0.84 |
| Third Ventricle | 48.2 | 49.2 | 45.7 | 49.5 | 2.6 0 | 0.55 |

[1]All data presented in this table represent the least-square means and pooled standard error of the means, significance was set determined at $p = 0.05$, with trends accepted at $0.05 < p < 0.10$. Number of pigs per dietary treatment was as follows: CON ($n = 10$), LOW ($n = 9$), MOD ($n = 9$), HIGH ($n = 8$).

**Supplemental Table 2**. Dietary sialyllactose does not influence relative brain volumes[1]

| *Region of Interest, (% total brain volume)* | **CON** | **LOW** | **MOD** | **HIGH** | **Pooled-SEM** | ***p*-Value** |
|---|---|---|---|---|---|---|
| Grey Matter | 56.39 | 56.51 | 57.59 | 56.88 | 0.811 | 0.68 |
| White Matter | 26.58 | 26.59 | 26.34 | 26.83 | 0.569 | 0.94 |
| Cerebrospinal Fluid | 9.45 | 10.86 | 10.83 | 11.84 | 1.168 | 0.30 |
| Caudate | 0.75 | 0.78 | 0.78 | 0.77 | 0.020 | 0.19 |
| Cerebellum | 9.48 | 9.49 | 9.7 | 9.78 | 0.186 | 0.27 |
| Cerebral Aqueduct | 0.07 | 0.08 | 0.07 | 0.07 | 0.002 | 0.12 |
| Corpus Callosum | 0.61 | 0.61 | 0.60 | 0.61 | 0.010 | 0.46 |
| Fourth Ventricle | 0.08 | 0.09 | 0.08 | 0.08 | 0.004 | 0.13 |
| Hypothalamus | 0.39 | 0.41 | 0.40 | 0.40 | 0.010 | 0.30 |
| Internal Capsule | 2.42 | 2.44 | 2.4 | 2.45 | 0.041 | 0.76 |
| Lateral Ventricle | 0.77 | 0.73 | 0.84 | 0.81 | 0.042 | 0.22 |
| Left Cortex | 26.00 | 26.69 | 26.76 | 26.72 | 0.243 | 0.06 |
| Left Hippocampus | 0.76 | 0.75 | 0.76 | 0.75 | 0.012 | 0.75 |
| Medulla | 2.81 | 2.81 | 2.86 | 2.96 | 0.053 | 0.19 |
| Midbrain | 2.96 | 3.00 | 2.93 | 3.02 | 0.050 | 0.61 |
| Olfactory Bulb | 3.82 | 3.88 | 3.74 | 3.84 | 0.105 | 0.55 |
| Pons | 1.78 | 1.77 | 1.74 | 1.80 | 0.030 | 0.45 |
| Putamen-Globus Pallidus | 0.65 | 0.63 | 0.64 | 0.64 | 0.010 | 0.79 |
| Right Cortex | 27.14 | 27.04 | 27.67 | 27.58 | 0.275 | 0.24 |
| Right Hippocampus | 0.78 | 0.77 | 0.78 | 0.77 | 0.013 | 0.96 |
| Thalamus | 2.56 | 2.58 | 2.57 | 2.60 | 0.032 | 0.66 |
| Third Ventricle | 0.08 | 0.08 | 0.07 | 0.08 | 0.004 | 0.31 |

[1]All data presented in this table represent the least-square means and pooled standard error of the means, significance was set at $p$ = 0.05, with trends accepted at 0.05 < $p$ < 0.10. Number of pigs per dietary treatment was as follows: CON ($n$ = 10), LOW ($n$ = 9), MOD ($n$ = 9), HIGH ($n$ = 8).

**Supplemental Table 3.** Dietary SL does not affect single-voxel spectroscopy measures[1]

| *Metabolite, ppm* | **CON** | **LOW** | **MOD** | **HIGH** | **Pooled SEM** | ***p*-Value** |
|---|---|---|---|---|---|---|
| Phospho-creatine | 2.283 | 2.596 | 2.051 | 2.122 | 0.285 | 0.38 |
| Glutamate | 2.725 | 2.583 | 2.203 | 2.350 | 0.166 | 0.07 |
| Glutatione | 0.965 | 1.061 | 0.969 | 1.136 | 0.070 | 0.19 |
| Myo-inositol | 5.220 | 5.315 | 5.325 | 5.205 | 0.305 | 0.98 |
| Scyllo-inositol | 0.309 | 0.301 | 0.283 | 0.307 | 0.022 | 0.77 |
| N-acetylaspartate | 4.242 | 4.462 | 4.126 | 4.546 | 0.226 | 0.31 |

[1]All data presented in this table represent the least-square means and pooled standard error of the means, significance was set at $p = 0.05$, with trends accepted at $0.05 < p < 0.10$. Number of pigs per dietary treatment are as follows: CON ($n = 10$), LOW ($n = 9$), MOD ($n = 7$), HIGH ($n = 7$).

**Supplemental Figure 1**. LOW and MOD pigs exhibit differences in tract-based spatial statistics

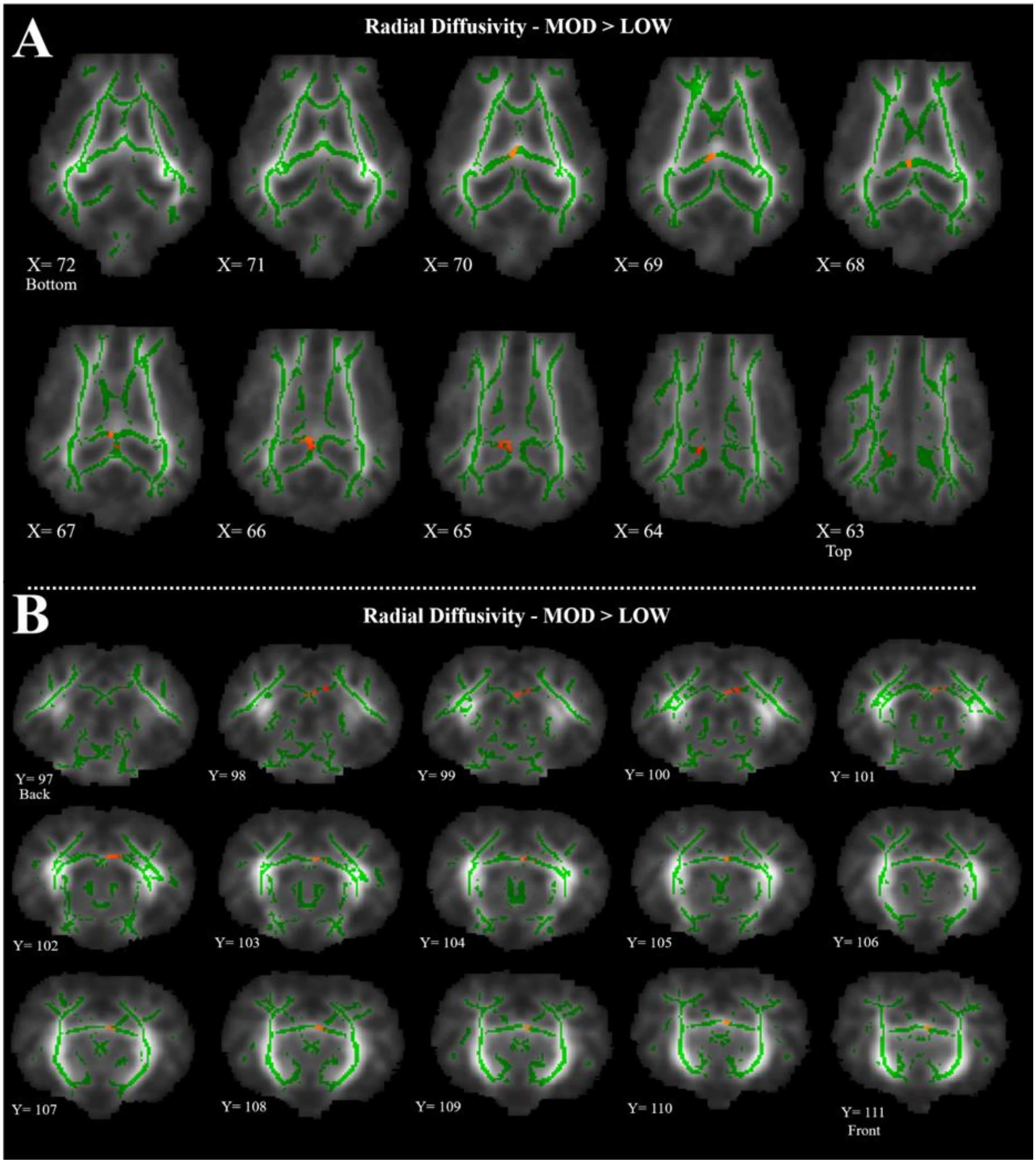


**Figure 3.** Tract-based spatial statistics (TBSS) illustrating differences in radial diffusivity (RD) between LOW and MOD dietary sialyllactose (SL) pigs. Pigs provided the MOD dietary SL treatment exhibited higher ($p < 0.05$) rates of radial diffusivity in the left hemisphere corpus callosum, when compared with LOW dietary SL pigs. The images generated from TBSS are and average of all LOW and MOD dietary SL pigs, green lines indicate regions in which all pigs exhibited white matter voxels. (**A**) Axial slices, with varying X-coordinates and static Y = 120 and Z = 76 coordinates, determined using University of Illinois Pig Brain Atlas [14]. (**B**) Coronal slices, with varying Y-coordinates and static X = 73 and Z = 87 coordinates, determined using the University of Illinois Pig Brain Atlas. Dark red and light red colors indicate degree of statistical differences from $p = 0.05$ to $p < 0.01$, respectively.

**Supplemental Figure 2**. LOW and MOD pigs exhibit differences in grey and white matter concentrations

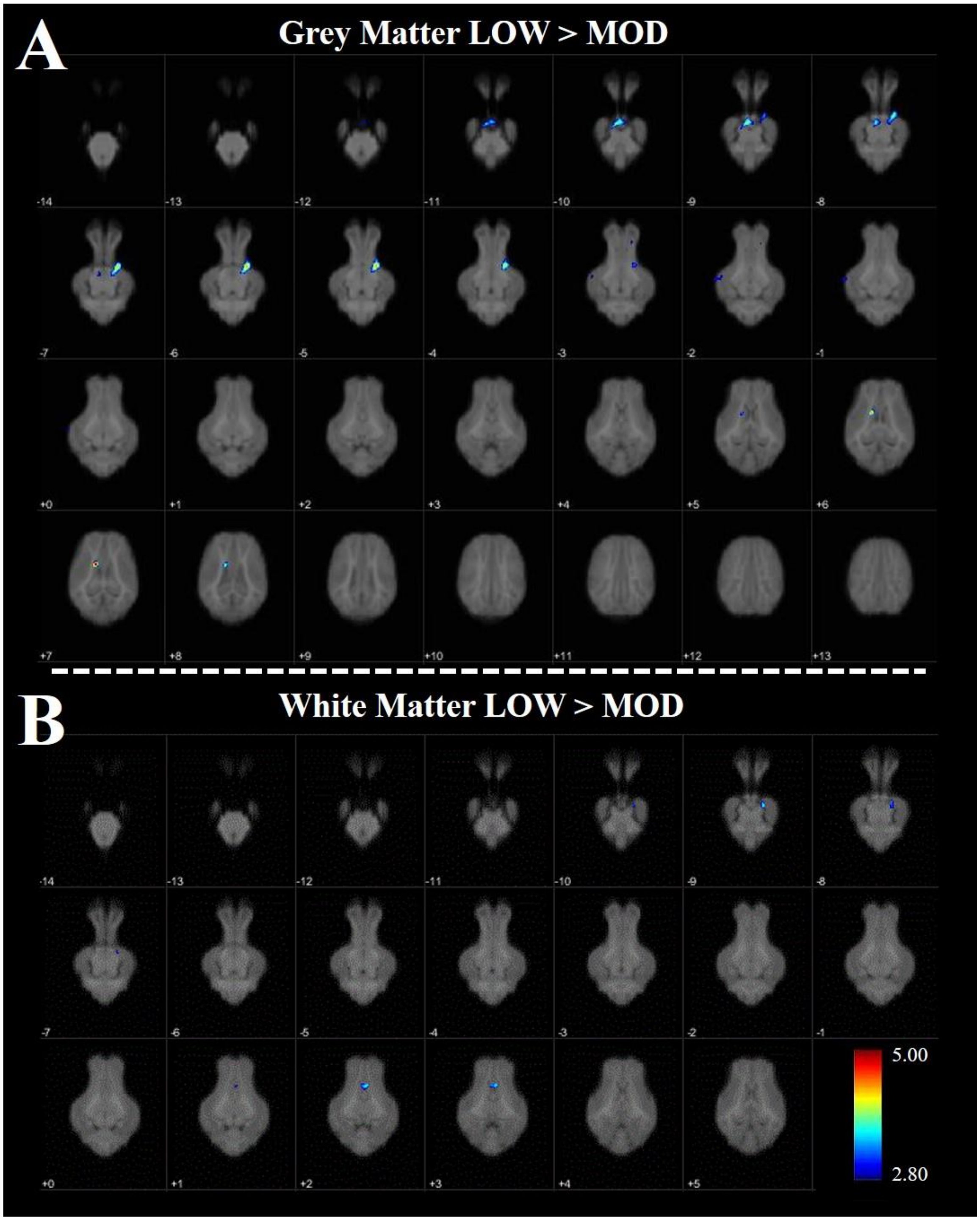


**Figure 4.** Voxel-based morphometry (VBM) heat maps illustrating grey matter tissue concentration differences between LOW and MOD dietary sialyllactose (SL) pigs. The colored bar indicates pseudo-$t$ statistics, used to determine the $p$-uncorrected statistics provided in Table 1. **(A)** Shown above are areas in which LOW dietary SL pigs exhibited greater ($p < 0.01$) concentrations of grey matter when compared with MOD dietary SL pigs. **(B)** Shown above are areas in which LOW dietary SL pigs exhibited greater ($p < 0.01$) concentrations of white matter when compared with MOD dietary SL pigs.
